# A Social Media–Promoted Educational Community of Joint Replacement Patients Using the WeChat App: Survey Study

**DOI:** 10.2196/18763

**Published:** 2021-03-18

**Authors:** Xianzuo Zhang, Xiaoxuan Chen, Nikolaos Kourkoumelis, Ran Gao, Guoyuan Li, Chen Zhu

**Affiliations:** 1 Department of Orthopedics The First Affiliated Hospital, Division of Life Sciences and Medicine University of Science and Technology of China Hefei China; 2 College of Chemistry and Chemical Engineering Xiamen University Xiamen China; 3 Department of Medical Physics School of Health Sciences University of Ioannina Ioannina Greece; 4 Department of Applied Psychology School of Humanities Guangdong Peizheng College Guangzhou China

**Keywords:** WeChat, social media, arthroplasty, perioperative education, patient satisfaction

## Abstract

**Background:**

Much effort has been made to optimize the results of total hip arthroplasty and total knee arthroplasty. With the rapid growth of social media use, mobile apps, such as WeChat, have been considered for improving outcomes and patient satisfaction after total hip arthroplasty and total knee arthroplasty.

**Objective:**

We aimed to evaluate the effectiveness of a WeChat-based community as an intervention for overall patient satisfaction.

**Methods:**

The study was conducted among discharged in-hospital patients who received hip or knee procedures in the First Affiliated Hospital of the University of Science and Technology of China from April 2019 to January 2020. An educational online social community was constructed with the WeChat app. Participants willing to join the community were enrolled in a WeChat group and received 3 months of intervention and follow-up. Those who were not willing to use the account were included in a control group and received routine publicity via telephone, mail, and brochures. The Danish Health and Medicine Authority patient satisfaction questionnaire was used to score perioperative patient education and overall satisfaction. The contents in the group chat were analyzed using natural language processing tools.

**Results:**

A total of 3428 patients were enrolled in the study, including 2292 in the WeChat group and 1236 in the control group. Participants in the WeChat group had higher overall satisfaction scores than those in the control group (mean 8.48, SD 1.12 vs mean 6.66, SD 1.80, *P*<.001). The difference between the two groups was significant for primary surgery based on subgroup stratification. To control confounding factors and explore the effects of WeChat participation as a mediating variable between perioperative patient education and overall satisfaction, hierarchical regression was utilized. An interpatient interaction model was found in the community group chat, and it contributed to overall satisfaction. Patients in the group with more interpatient interactions were more likely to have better overall satisfaction.

**Conclusions:**

The social media–promoted educational community using WeChat was effective among joint replacement patients. Provision of more perioperative education is associated with more active patient participation in the community and therefore more patient satisfaction in terms of the overall joint procedure. Community group chat could facilitate interactions among patients and contribute to overall satisfaction.

## Introduction

Joint diseases of the lower limb extremities influence the quality of life of elderly people and are becoming increasingly frequent owing to the rising average life expectancy. To date, total hip arthroplasty and total knee arthroplasty have been the most successful surgical treatments for end-stage arthritis. Considerable progress has been made to optimize the end-to-end process by improving the surgical technique, rehabilitation, and perioperative care. Although the length of hospital stay has been shortened in recent years [1], rehabilitation remains a long-term procedure and a physiopsychological challenge for patients who have undergone arthroplasty. Continuous rehabilitation guidance has proved its efficacy during long-term follow-up [2-4]. However, most of the rehabilitation advice in China is given during hospital stay rather than in a community center. Since patients often have limited awareness and poor compliance after discharge, educational follow-up needs to be strengthened further.

Perioperative education increases patient satisfaction [5,6], but routine guidance via telephone and mail can be laborious and challenging for elderly patients owing to age, educational level, and cognitive ability, among other implementation issues like the time management of physicians’ firms. Novel mobile health (mHealth) platforms allow patients to communicate remotely with their health care providers. Some systems facilitate online sharing of smartphone photographs in a secure and Health Insurance Portability and Accountability Act compliant manner [7], potentially allowing for simple and rapid postoperative rehabilitation monitoring. However, building a professional mHealth platform is resource consuming and typically inefficient because patient engagement is persistently low, especially in large user groups [8].

Nevertheless, with so many people using social media every day, there is a great opportunity for mHealth initiatives to positively influence health attitudes and behaviors among large groups of people [9]. Interventions involving various media, such as radio, television, and the internet [10], have already been utilized for mass outreach health campaigns [11]. Because online social networks have several advantages, such as a large audience, increased user engagement, and high retention of contacts [12], they can strongly promote healthy behavioral changes [13]. In fact, social media approaches have already been pilot tested with promising outcomes [14,15]. Yet, studies on larger population data are pending.

WeChat (the Chinese version is *Weixin*) is the most popular instant-messaging app in China, which has been created by China’s largest internet company, Tencent [16]. It is used in more than 200 countries [17], with approximately 1 billion active accounts in the first quarter of last year [18]. WeChat is a messaging and social media app where people of all ages and professions can either collaborate or just share information and messages [16]. WeChat was previously used with success in a health education program to improve malaria health literacy among Chinese expatriates and was proven to be effective, sustainable, feasible, and well accepted [17]. Similar time and cost-effectiveness were observed during the WeChat follow-up [19]. In a second example, WeChat interventions improved patient compliance and reduced the treatment duration of orthodontic treatment [2]. In this study, we scrutinized the hypothesis that social media can enhance the educational interaction between patients with joint replacement and physicians.

## Methods

### Study Design and Sample Selection

We performed a nonrandomized controlled study in a single-institute in-hospital population from April 2019 to January 2020. Patients receiving joint replacement surgeries in the First Affiliated Hospital of the University of Science and Technology of China were recommended to join the WeChat campaign on admission. The participants who enrolled (1) received elective lower extremity arthroplasty surgery in our joint center, (2) were willing to receive relative health and rehabilitative education, and (3) had WeChat app–compatible smartphones. Patients excluded from the study were either unwilling to participate or received emergency surgery. Participants who joined our WeChat campaign were added to a WeChat group, received at least 3 months of online follow-up, and filled a satisfaction survey. Patients not willing to join were informed about the study and were included in a control group. These patients received routine follow-up through telephone calls, phone messages, and face-to-face appointments at the clinics. They were also asked to fill a written questionnaire form on re-examination.

### WeChat Campaign Construction

**Figure 1:**
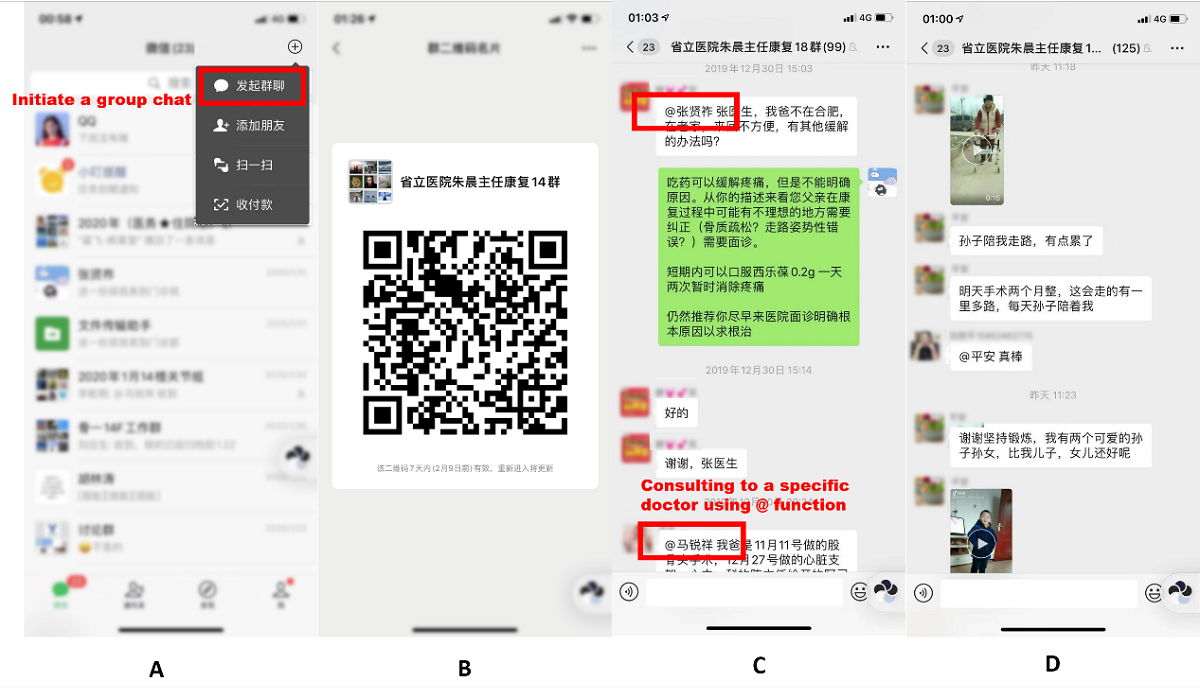
Creation of a chat group using the smartphone social media app WeChat. (A) Initiation of a group chat with personal contacts. (B) Generation of a QR code for patient invitation. (C) Consultation with a specific doctor/nurse/therapist using the @ function. (D). Sharing of treatment and rehabilitation experiences with video clips. Note that patient profile photos and names are hidden.

The group chat was created using the built-in function of the WeChat app (Figure 1A). The app allows individuals to create multiple user dialogues among their contacts. The initial group chat was built among orthopedic surgeons (n=6), ward and clinic nurses (n=3), and physical therapists (n=3). A QR code was generated for each group automatically (Figure 1B). Thereafter, the QR codes were shared with the patients in order to join the group before leaving the hospital. The maximum number of group members is 500. Therefore, a new chat group was created monthly to facilitate maintenance. The medical staff participating in the group used their real names. Patients were encouraged to use real names, although they were reassigned in-hospital IDs by the group administrator (CZ). Health advice, including clinical and hospital instructions, rehabilitation guides, and general health education, were released weekly by the doctors, nurses, and therapists. Patients raised point-to-point questions to doctors using the @ function (Figure 1C). They were also encouraged to complain or share their surgery and rehabilitation experiences (Figure 1D).

### Interventions and Measures

Demographic data, such as gender, age, and educational level, were collected upon hospital admission. The patients in both groups received the same protocol treatment, rehabilitation, and education during hospital stay. After discharge, the interventions were provided through the WeChat app and routine publicities, including verbal publicity, telephone interviews, and a two-page take-home brochure. The patients were asked to fill a perioperative education and satisfaction questionnaire online or on re-examination. The questionnaire was originally developed and pilot tested by the Danish Health and Medicine Authority (Danish Health and Medicine Authority 2006) (Multimedia Appendix 1) [1]. Outcomes, including information given and overall satisfaction, were measured on a numerical rating scale from 0 (not satisfied at all) to 10 (best possible satisfaction).

### WeChat Dialogue Analysis

The group chat dialogues were recorded and encrypted in the WeChat app (WeChat 7.0.10 for iOS, Tencent Co, Ltd). The records were exported in CSV format to a database using Sync tools (I4Assistant, v7.98.12, Shenzhen Waip Information Technology Co, Ltd). Information regarding group number, message timestamp, user ID (identifier), message type, and content was read from the database. The users were marked as P (patient) and D (doctor) according to the user IDs. Patients who had more than 10 single messages or three conversations were characterized as “active participants.” The message contents were recorded in plain text, figures, and videos. Voice messages were converted to text via the WeChat built-in speech recognition function. External weblinks and system messages were dropped. Text processing and analysis was preformed using Python v3.7.0 (Python Software Foundation). For English content, a blank character was used as a mandatory tokenizer. For Chinese content, we imported the external library Jieba [20] for word segmentation. The word frequency was calculated among the dialogues, and the conversation contents were clustered into different groups. A word cloud was drawn using the external library wordcloud 1.3.3 [21], and the additional Python libraries xlrd, xlwt, math, and matplotlib were used for the analysis and the corresponding plots. The drawing source code is provided in Multimedia Appendix 2.

Communication between patients was analyzed using a patient interaction score. Specifically, the first message in every group chat was assigned an interaction score value of zero, and the score of the next message was increased by one if it originated from a different patient user. However, the score was forced to zero if the message was sent from medical staff. This parameter describes how intensive the communication is among different patients under the supervision and guidance of a doctor. The group activity was determined by activeness rates defined as the total score divided by the total number of messages. The flowchart of the process is given in Multimedia Appendix 3.

### Ethics Statement

The study was approved by the hospital institutional review board. All followed procedures were in accordance with the ethical standards of the institutional and national research committee and with the 1964 Helsinki declaration and its later amendments or comparable ethical standards. All participants gave verbal consent to participate in the study, and they were entitled to withdraw from the study whenever they wished and for whatever reason. Private data, such as telephone numbers and identification numbers, were deleted during data analysis.

### Statistical Analysis

Statistical analysis was carried out using SPSS Statistics version 22.0 (2013, SPSS Inc). For continuous variables, analysis of variance (ANOVA) and post-hoc analysis by Fisher least significant difference were applied to compare the means among the study groups. For categorical variables, logistic regression analysis and contingency tables were used. Spearman and Pearson analyses were performed to investigate monotonic and linear relationships, respectively. To control for confounding factors, propensity score methods with a multinominal logistic regression were utilized. The propensity score replaced all single covariates to adjust the effectiveness on patient satisfaction. A hierarchical regression model was used to test potential mediating or moderating effects. Data were expressed as mean (SD) for continuous variables or percentage of the total for categorical variables. A *P* value <.05 was considered statistically significant.

## Results

### Participant Characteristics and WeChat Activeness

**Figure 2:**
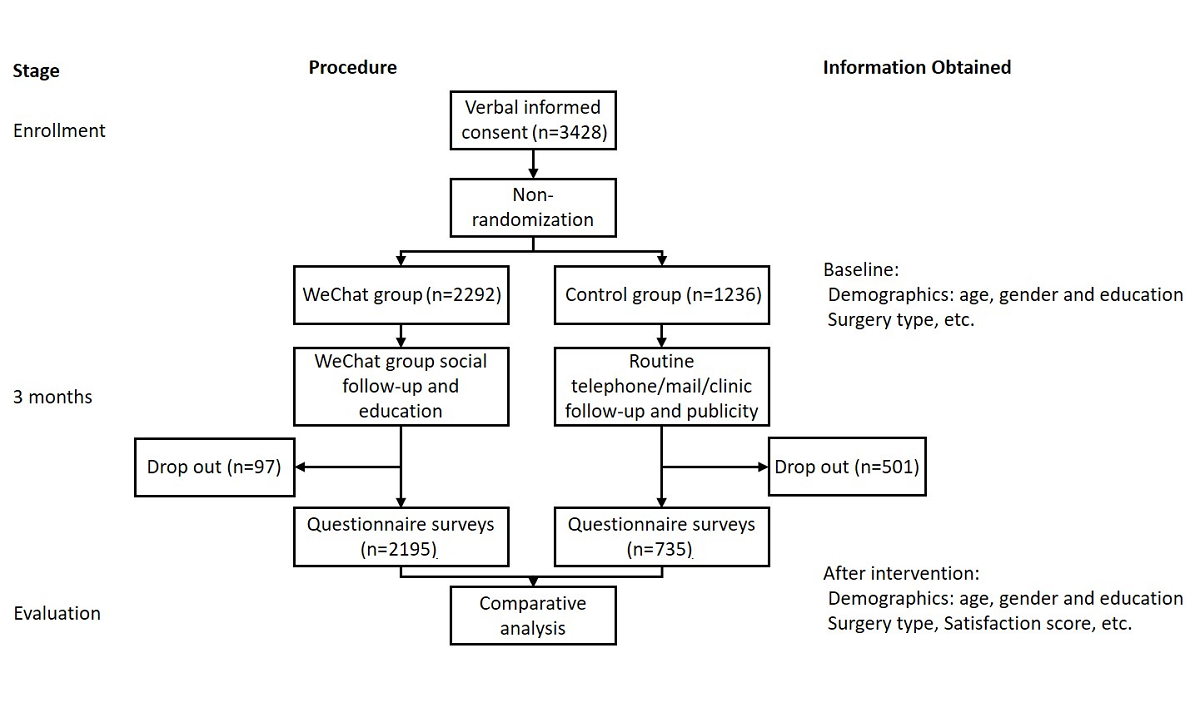
Flowchart of participation.

A total of 3428 patients were invited to participate in the study, including 2292 in the WeChat group and 1236 in the control group. Data were collected from 2930 participants (2195 in the WeChat group and 735 in the control group). After 3 months of intervention and follow-up, information was obtained and analyzed (Figure 2). The mean age of the enrolled participants was 62.19 years (SD 12.67 years). In total, 1973 (67.34%) females and 957 (32.66%) males were included in this survey. Additionally, 2327 (79.42%) individuals had basic education, 250 (8.53%) had medial education, and 353 (12.05%) had high education. A total of 1034 (35.29%) patients underwent total hip arthroplasty, 73 (2.49%) underwent hemiarthroplasty, 96 (3.28%) underwent total hip revision, 1589 (54.23%) underwent total knee arthroplasty, 91 (3.16%) underwent unicompartmental knee arthroplasty, and 47 (1.60%) underwent total knee revision. The baseline demographics (age, gender, and educational level) were not balanced between the two groups (Table 1). Patients who had more than 10 single messages or three conversations formed the group of “active participants.” We found 738 out of 2195 (33.62%) patients active in the WeChat group (ie, the majority were inactive in the group chats). Active patients were typically males, young, and educated. The differences between the control group and WeChat group in terms of demographics were all statistically significant (Table 1).

**Table 1:** Demographic characteristics of the control group and WeChat group (N=2930).

Demographic characteristic		Control group (n=735), n (%)	WeChat group (n=2195), n (%)				Chi-square (*df*)^a^		*P* value^a^
			Inactive (n=1457)	Active (n=738)	Total				
**Gender**						41.88 (2)		<.001	
	Male	223 (30.34)	422 (28.96)	312 (42.28)	734 (33.44)				
	Female	512 (69.66)	1035 (71.04)	426 (57.72)	1461 (66.56)				
**Age group (years)**						400.194 (2)		<.001	
	<65	339 (46.12)	543 (37.27)	605 (81.98)	1148 (52.30)				
	≥65	396 (53.88)	914 (62.73)	133 (18.02)	1047 (47.70)				
**Education level^b^**						482.017 (4)		<.001	
	Low	597 (81.22)	1286 (88.26)	444 (60.16)	1730 (78.82)				
	Medial	87 (11.84)	123 (8.44)	40 (5.42)	163 (7.43)				
	High	51 (6.94)	48 (3.29)	254 (34.42)	302 (13.76)				
**Surgery type**						379.55 (10)		<.001	
	THA^c^	162 (22.04)	410 (28.14)	462 (62.60)	872 (39.73)				
	HA^d^	23 (3.13)	43 (2.95)	7 (0.95)	50 (2.28)				
	THR^e^	9 (1.22)	59 (4.05)	28 (3.79)	87 (3.96)				
	TKA^f^	488 (66.39)	886 (60.81)	215 (29.13)	1101 (50.16)				
	UKA^g^	41 (5.58)	29 (1.99)	21 (2.85)	50 (2.28)				
	TKR^h^	12 (1.63)	30 (2.06)	5 (0.67)	35 (1.60)				

^a^Chi-square and *P* values indicate differences between the control group, inactive group, and active group.

^b^Education level: low, compulsory education; medial, high school or equivalent; high, university/college or above.

^c^THA: total hip arthroplasty.

^d^HA: hemiarthroplasty.

^e^THR: total hip revision.

^f^TKA: total knee arthroplasty.

^g^UKA: unicompartmental knee arthroplasty.

^h^TKR: total knee revision.

### Overall Satisfaction Scores Between the Control Group and WeChat Group

**Figure 3:**
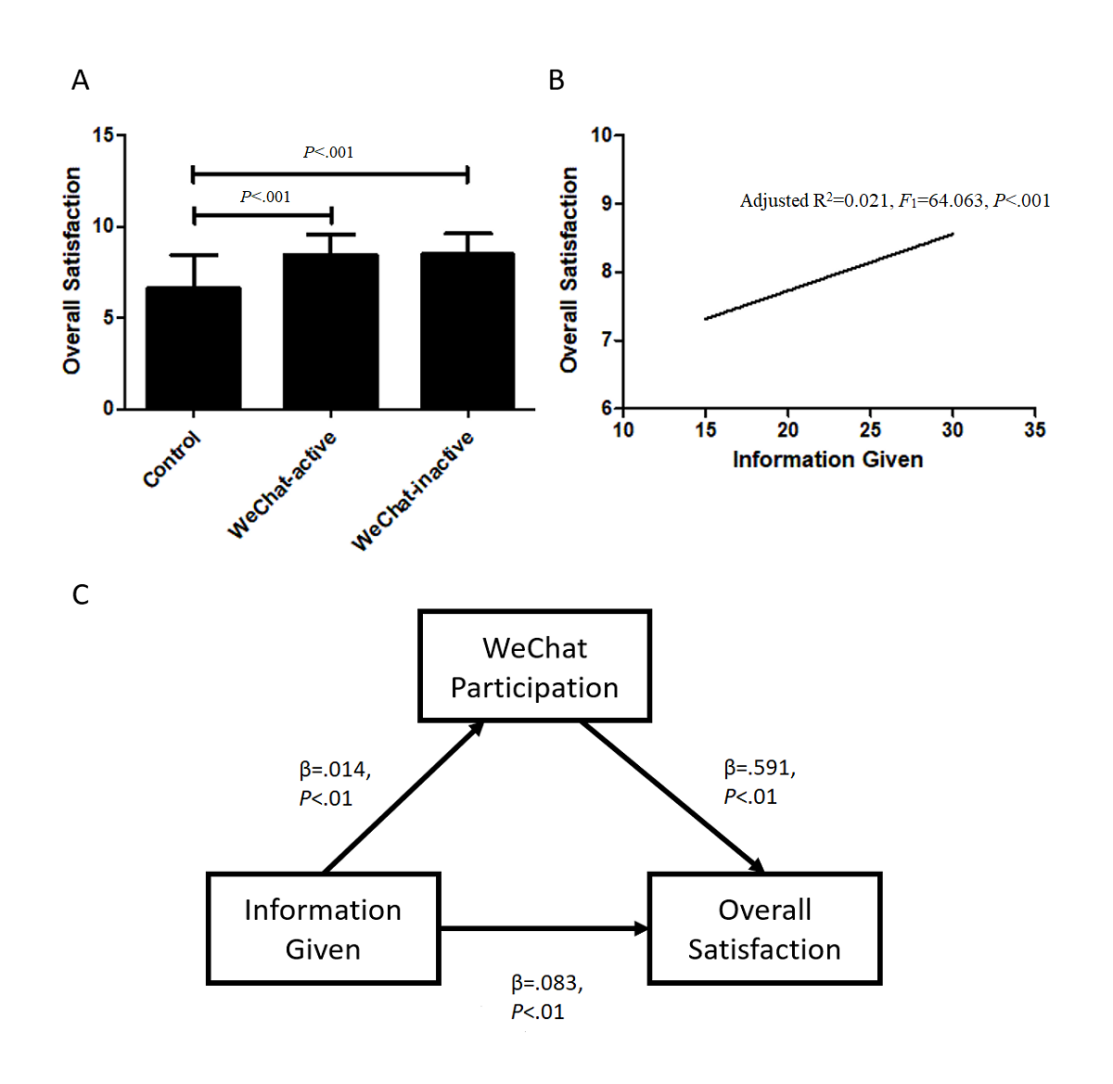
Data analysis from the Danish Health and Medicine Authority 2006 questionnaire. (A) Histogram shows higher overall satisfaction in active patients and inactive patients in the WeChat group than in the control group. (B) Linear regression between overall satisfaction and information given. (C) The mediated effect model among information given, WeChat participation, and overall participation.

In general, patients in the WeChat group provided higher overall satisfaction scores than those in the control group (mean 8.48, SD 1.12 vs mean 6.66, SD 1.80; *P*<.001). Subgroup analysis supported this finding in both active (mean 8.47, SD 1.11 vs mean 6.66, SD 1.80; *P*<.001) and inactive patients (mean 8.49, SD 1.13 vs mean 6.66, SD 1.80; *P*<.001) (Figure 3A). Since the baseline demographics were not balanced, the correlations between overall satisfaction and gender, age, education, and surgery type were tested. It was found that overall satisfaction was not related to gender (*P*=.07), age (*P*=.21), and education level (*P*=.63), but was significantly related to surgery type (*P*<.001). Therefore, a stratified analysis was performed in order to eliminate confounding bias (Table 2). It was found that patients receiving primary lower extremity arthroplasty reported significantly higher overall satisfaction scores in the WeChat group than those in the control group. The difference between groups was not significant in patients receiving revision surgery.

**Table 2:** Total satisfaction score in the control group and WeChat group by age and surgery type (N=2930).

Surgery type and group		Value, n	Score, mean (SD)	*t* (*df*)	*P* value
**THA^a^**				−12.525 (181.914)	<.001
	Control	162	6.51 (1.94)		
	WeChat	872	8.47 (1.13)		
**HA^b^**				−4.805 (28.914)	<.001
	Control	23	6.52 (1.95)		
	WeChat	50	8.62 (1.12)		
**THR^c^**				−1.514 (9.161)	.16
	Control	9	7.78 (1.39)		
	WeChat	87	8.51 (1.15)		
**TKA^d^**				−21.444 (667.963)	<.001
	Control	488	6.65 (1.74)		
	WeChat	1101	8.49 (1.11)		
**UKA^e^**				−4.337 (70.902)	<.001
	Control	41	7.02 (1.65)		
	WeChat	50	8.36 (1.19)		
**TKR^f^**				−2.061 (13.186)	.06
	Control	12	7.17 (2.21)		
	WeChat	35	8.54 (1.17)		

^a^THA: total hip arthroplasty.

^b^HA: hemiarthroplasty.

^c^THR: total hip revision.

^d^TKA: total knee arthroplasty.

^e^UKA: unicompartmental knee arthroplasty.

^f^TKR: total knee revision.

### Analysis of Perioperative Education and Overall Satisfaction in Patients Receiving Arthroplasty

Perioperative education was measured using *information given* scores acquired from the Danish Health and Medicine Authority satisfaction questionnaire. A significant linear correlation was found between information given and overall satisfaction scores (adjusted R^2^=0.021, *F*_1_=64.063, *P*<.001) (Figure 3B). Owing to the small effect size (β=.08), we constructed and tested a causal mediation effect model to illustrate the relationship among perioperative education, WeChat campaign participation, and overall patient satisfaction. The percentage of the mediated effect of WeChat participation between perioperative education and overall patient satisfaction was 10.75% (Figure 3C).

### WeChat Group Conversation Analysis

**Figure 4:**
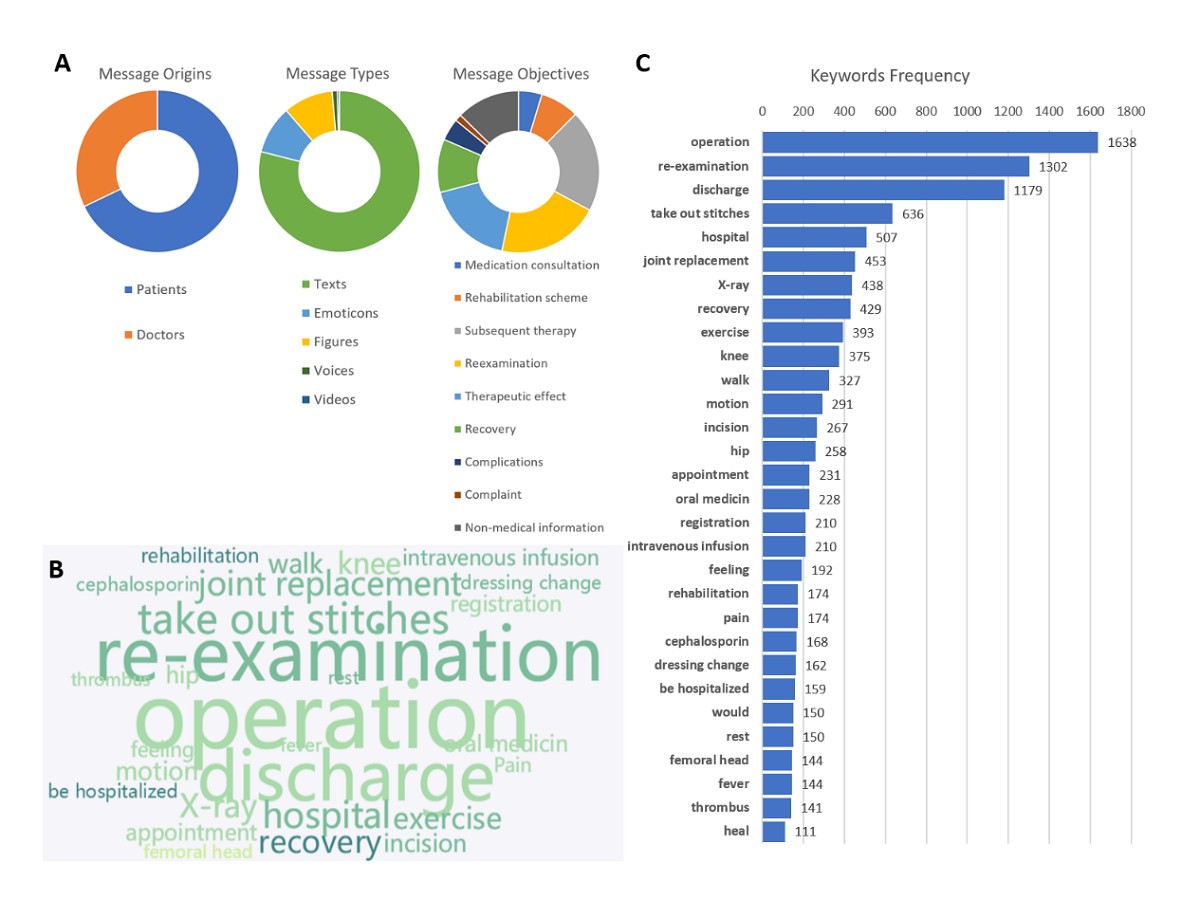
Text analysis of group chat records. (A) Source, classification, and purpose of the messages. (B) Word cloud of the most frequently mentioned words in the group chats. (C) Top 30 medical words that appeared in the group chats.

From April 2019 to March 2020, a total of 23,088 messages were recorded in eight group chats. There were 7422 (32.15%) messages from doctors and 15,666 (67.85%) messages from patients. The types of messages were as follows: text, 18,231 (78.96%); emoticons, 2226 (9.64%); figures, 2298 (9.95%); voice messages, 240 (1.04%); and videos, 93 (0.40%). The purposes of messages were as follows: medication consultation, 867 (4.69%); rehabilitation scheme, 1425 (7.72%); subsequent therapy, 3782 (20.48%); re-examination, 3769 (20.41%); reflect therapeutic effects, 3249 (17.59%); report recovery progress, 1970 (10.67%); inquire about operation related complications, 816 (4.42%); complaints, 249 (1.35%); and nonmedical information, 2344 (12.69%) (Figure 4A). The content of messages was transformed into plain text, and the resulting unstructured text of 315,900 Chinese characters was tokenized using the Python library Jieba. The top 100 words were extracted for further analysis. Nonmedical words were screened out manually in this step. The top watched words included operation, re-examination, discharge, take out stitches, and hospital. The top 30 words with the highest frequency are shown in the form of a word cloud in Figure 4B and 4C. A positive side-effect of our approach was that six hip joint dislocations and 11 surgical-site infections were detected and reported early in the WeChat patient cohort.

**Figure 5:**
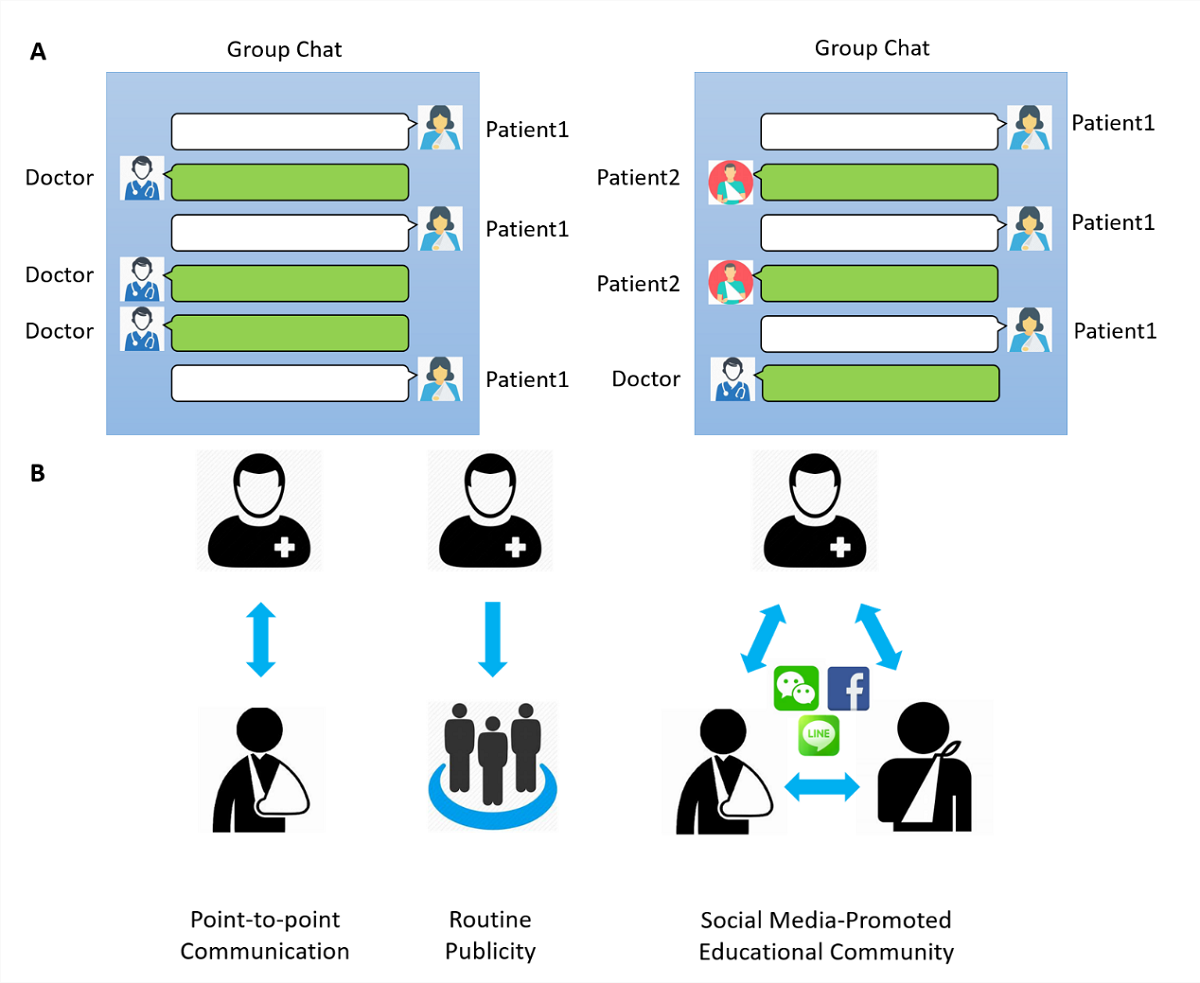
Doctor-patient communication and health educational model. (A) Left: doctor-patient conversation; right: patient-patient conversation in the group chat. (B) Different interaction models between doctors and patients. Left: one-to-one interactions between doctors and patients; middle: unilateral publicity from doctors to patients; right: social media–promoted multidirectional educational community among doctors and patients.

**Figure 6:**
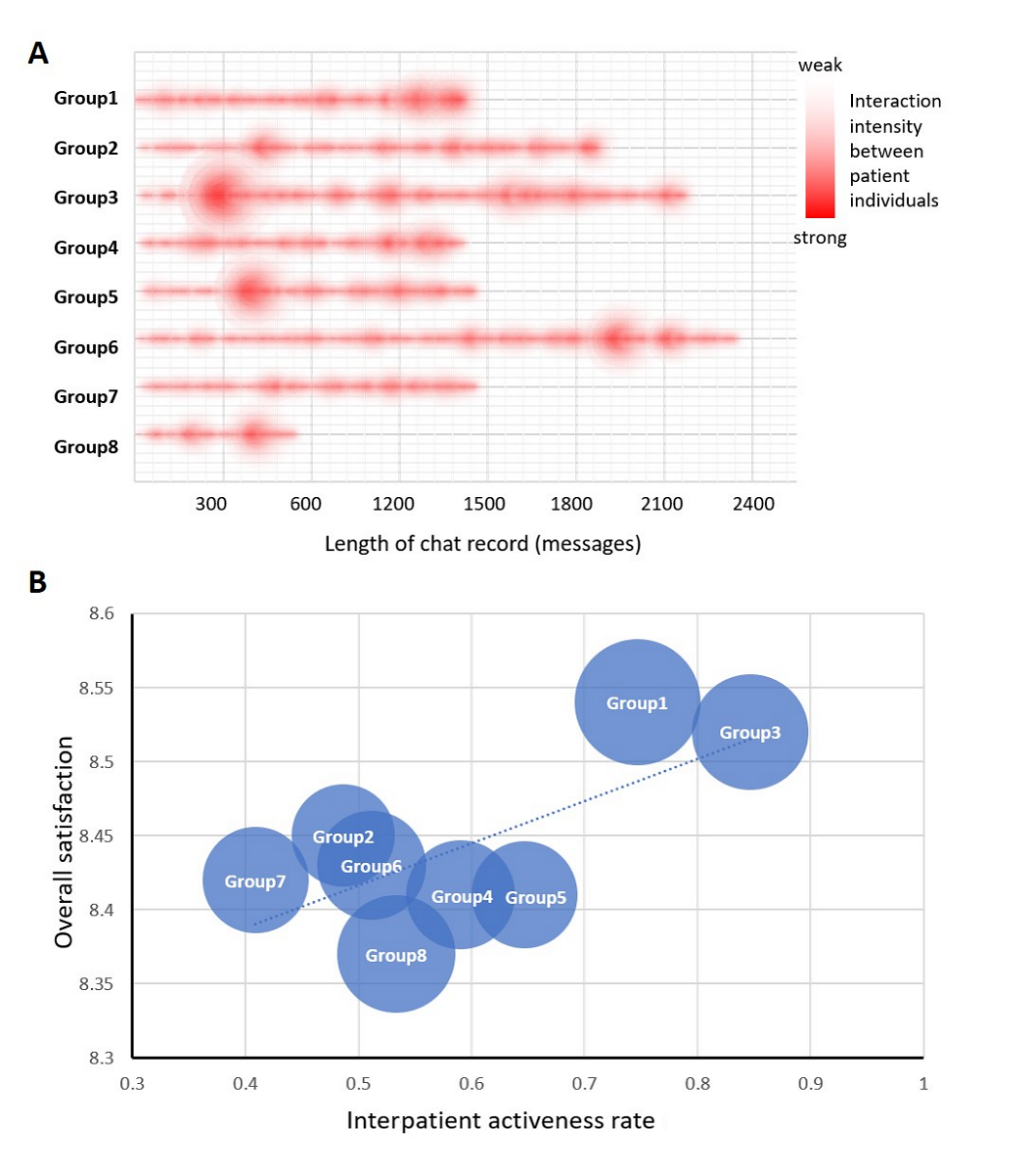
The patient-patient interaction intensity. (A) A heatmap reflecting the interaction intensity in different portions of the group chats. Abscissa: total length of the entire group chat. (B) Balloon map showing the overall satisfaction and interpatient activeness rate in different groups. The diameter of each circle is determined by the number of group members.

Apart from one-to-one conversations between doctors and patients, conversations between patients were analyzed with interaction scores and activity rates (Figure 5). The interaction intensity is shown in a heatmap in Figure 6A. Among the eight temporally continuous group chats, the highest interaction score (35 points) was found in Group 3. We also found that patients in groups with higher activeness rates (Group 1 and Group 3) had better overall satisfaction (Figure 6B). Linear regression of overall satisfaction over the activeness rate was attempted, but it resulted in nonsignificance (adjusted R^2^=0.28, *F*_1_=3.36, *P*=.13).

## Discussion

### Principal Findings

In addition to surgical success, long-term postoperative rehabilitation is essential for better outcomes after joint replacement surgery. Inadequate patient compliance may result in unsatisfactory joint function and poor quality of life in the course of home-based rehabilitation. Persistent medical intervention after discharge is important for joint function recovery and patient satisfaction [2,22]. Standard care for the rehabilitation of knee conditions involves exercise programs and information provision. Current methods of rehabilitation struggle to keep up with the large volumes of patients and the lengthy treatment required for recovery. Herein, we described a novel smartphone-based community approach for rehabilitation guidance and medical support in postoperative patients. This nonrandomized study showed that a social media–enhanced educational community is an effective approach to promote patients’ satisfaction with primary arthroplasty.

Social media can influence lifestyle, including health behavior [23], especially for large groups of people [24]. In our study, 2292 out of 3428 (66.86%) patients who underwent joint replacement surgery were willing to participate in the WeChat-based community to evaluate the quality and the effect of postoperative care via the internet. Compared with professional mHealth platforms, the social media WeChat-based community has far more active users. It is estimated that online social networks account for approximately 27.18% of all the time people spend online [3], and 55.2% of WeChat users check the WeChat status over 10 times per day, while 25.0% of users check the status over 30 times per day [25]. Therefore, we hypothesized that medical information can be delivered more efficiently in this way.

In general, participants in the WeChat group had higher overall satisfaction scores (mean 8.48, SD 1.12) than those in the control group (mean 6.66, SD 1.80), which is consistent with previous studies [3,4,19]. The smartphone WeChat app has been found to be a viable option for follow-up in discharged patients with head and neck tumors [19]. Therefore, the doctor-led follow-up model has the potential to establish a good physician-patient relationship by enhancing dynamic communication and providing individual health instructions. Therapeutic guidance and intervention via WeChat also improved rehabilitation after total hip arthroplasty and promoted the recovery of joint function in patients [3]. The platform helps patients to comprehend disease knowledge and rehabilitation exercise methods, promoting recovery [4]. Using stratified analysis, we observed the significant satisfaction advantages of WeChat in patients receiving primary lower limb extremity arthroplasty, while the effect was less notable in patients receiving revision surgeries. Despite the fact that the long-term quality of life is poorer after revision surgery than after primary surgery [26], the patient satisfaction scores were relatively high in the WeChat and control groups during hospitalization, probably because patients who had a previous operation history were better informed and less anxious.

Perioperative patient education is widely used to inform patients and relatives about various aspects of the upcoming operation and to motivate patients to be active participants [6]. However, the effects of perioperative patient education on surgical outcomes remain to be considered. Louw et al found that adding a brief 30-minute pain neuroscience education session to a traditional preoperative total knee arthroplasty education program did not result in any significant improvements, except for patient satisfaction [6]. Perioperative patient education might increase patient satisfaction by communicating realistic expectations to the patients [5]. In this study, a linear regression between overall patient satisfaction and information given was observed as an indicator for perioperative patient education levels. Although statistically significant (*P*<.001), the effect size of perioperative patient education on overall satisfaction was small (β=.08). It is most likely that WeChat participation mediates a perioperative patient education effect on patient satisfaction (ie, the more information given, the more participation is expected in the community interaction).

An important question is “how does WeChat participation and activeness influence patient satisfaction?” This study showed that although patients in the WeChat group had higher overall satisfaction scores than those in the control group, the scores between active and inactive patients were indifferent (Figure 3A). It was doubted whether this difference came from the selection bias due to the nonrandomized study design. However, on testing the baseline demographics, the difference remained insignificant between the control and WeChat groups. Instead, young and highly educated participants were among WeChat active users in contrast to inactive users. We believe that inactive users, although not participating in the group chat, still received information and social support from the experience shared by other active users. A series of reports has proven the positive effect of social support on the outcomes of lower limb extremity arthroplasty [27-30]. In studies of patients with hip fractures, a significant relationship was also identified between social support and lower limb functional activity [31]. Sveikata et al demonstrated better postoperative functional results 12 months after total knee arthroplasty in patients who had better social support [32].

Added social support stems from the interpatient interaction in the group chats. In addition to the traditional doctor-led follow-up models, medical doctors typically communicate with a single patient or address the public unilaterally. However, this new social media approach enables patients to play an active role in the network (Figure 6). Our study showed that patients in groups with more interpatient interaction were more satisfied with their overall treatment. The structural support may provide individuals with self-respect and motivation [28] for more appropriate health decisions [33]. Having a large amount of structural support from group chat members may also mean greater access to sources of information, which can increase the likelihood of having access to accurate and relevant information sources [28]. Functional support, on the other hand, may have a positive effect on health decision-making during periods of distress [30]. When an individual perceives high levels of social support, he or she may reassess a stressful event as less worrisome and become more capable to face a disease [11]. Therefore, it is important to know about the rehabilitation experience and to receive suggestions from other patients during recovery.

The effectiveness of medical interventions involving smartphone apps compared with those involving the internet has been debated, and the latter approaches have been suggested as being more accessible and convenient for patients [34], although a more recent report found smartphone apps to be more efficient and effective for online health care services [35], possibly due to the tremendous growth of smartphone usage. In the context of physical activity, it was found that over half of the controlled trials of web-based interventions reported positive behavioral outcomes [36]. Furthermore, a web-based intervention was found to be useful in delivering standard care for the rehabilitation of knee conditions [15]. However, poor adherence is a common problem with web-based approaches [15,37]. On the other hand, the most effective interventions are interactive and flexible, thereby allowing patients to select information that is relevant to their medical conditions and suitable at a particular point in time [37]. Facebook has been already used as a tool to promote public health and educational health services [38,39], while Twitter has been, to some extent, a possible alternative [40].

To date, social media usage is still controversial owing to privacy, security, confidentiality, and liability issues [15]. According to laws and regulations, communication between health care providers and patients must comply with current data security and privacy legislation. The presented social media community approach has the legal risk of leaking private patient information in an open group chat. Health care–centric discussions must ensure trusted moderation and storage facilities. We have to also pay attention to avoiding the possibility of spreading fake news and wrong interventions [41]. The medical staff members in the group therefore have the responsibility of community supervision.

This study has some limitations. First, it was a single-institute nonrandomized controlled study. Although propensity score methods were used and stratified analysis was performed, the conclusions were still limited to a small data set. Second, it was a retrospective study, and the baseline data (eg, education level) of the patients were not consistent between the different groups. Some patients may refuse to join the social media campaign simply because of electronic illiteracy. Additionally, a difference in satisfaction would be unsurprising. Further prospective balance-armed studies should be conducted to eliminate this issue. Third, patient satisfaction is a complex variable affected by multiple noncontrollable factors that were not evaluated (probably cannot be evaluated). Although this study is very preliminary, it is one of the few studies to analyze the interactions among patients for social media education after lower extremity joint replacement. Further investigations and clinical trials will bring more value to this field.

### Conclusions

The present social media–promoted educational community based on WeChat can improve overall satisfaction among hip and knee joint replacement patients. This new model of physician-patient community interaction can facilitate better postoperative rehabilitation.

## Appendix

Multimedia Appendix 1 The questions used in the Danish Health and Medicine Authority 2006 questionnaire.

Multimedia Appendix 2 The drawing source code to build the word cloud image.

Multimedia Appendix 3 The pseudocode flowchart to calculate the interaction score for messages.
