# Supplementary material for: A Social Media–Promoted Educational Community of Joint Replacement Patients Using the WeChat App: Survey Study
Source: JMIR Mhealth Uhealth. 2021 Mar 18;9(3):e18763. doi: 10.2196/18763 (PMC8088850; doi:10.2196/18763)
Supplement: Multimedia Appendix 1 [file mhealth_v9i3e18763_app1.docx]

**Multimedia Appendix 1.** The questions used in the [Danish Health and Medicine Authority 2006](https://www.ncbi.nlm.nih.gov/pmc/articles/PMC4750770/#C5) questionnaire.

| Content |
| --- |
| 1. Sex: Female / Male  2. Age: Years  3. What type of surgery did you have? (last surgery): Total knee replacement / Total hip replacement  4. How many days did you stay in hospital after your surgery? Days  5. Do you have any of the following conditions? (please mark the conditions you have): Diabetes, Cardiovascular disease, Lung disease, Rheumatoid arthritis  6. Did you receive a pre-admission leaflet? Yes / No  7. Did you receive any home care before surgery? Yes / No  8. If yes, how often did you receive home care before admission? Several times a day / Once a day / Several times a week / Once a week  9. How satisfied were you with the information you received before surgery ^a^?  10. How satisfied were you with the rehabilitation you received from the physiotherapists and the nursing staff during admission ^a^?  11. How satisfied were you with the pain treatment you received during admission ^a^?  12. How satisfied were you with the information you received during your admission ^a^?  13. Were you satisfied with the length of your stay in hospital? Yes / No, I would have preferred a longer stay / No, I would have preferred a shorter stay / Don’t know  14. How well-informed did you feel about the time after discharge ^a^?:  15. How satisfied were you with your discharge procedure ^a^?  16. How satisfied were you throughout the first few weeks after discharge ^a^? 0–10  17. How was your overall satisfaction with the entire process ^a^? |

^a^ (on a scale from 0 to 10, where 0 is not satisfied at all and 10 is best possible satisfaction)

Responses to 3 questions (questions 9, 12, and 14) concerning satisfaction with information given prior to admission, during the hospital stay, and after discharge (each on a scale of 0–10) were summed up, resulting in a satisfaction NRS total score from 0 to 30. The 3 items were merged into one item, and are reported in this paper as “information given”.
