# Supplementary figures and images for: A Social Media–Promoted Educational Community of Joint Replacement Patients Using the WeChat App: Survey Study
Source: JMIR Mhealth Uhealth. 2021 Mar 18;9(3):e18763. doi: 10.2196/18763 (PMC8088850; doi:10.2196/18763)

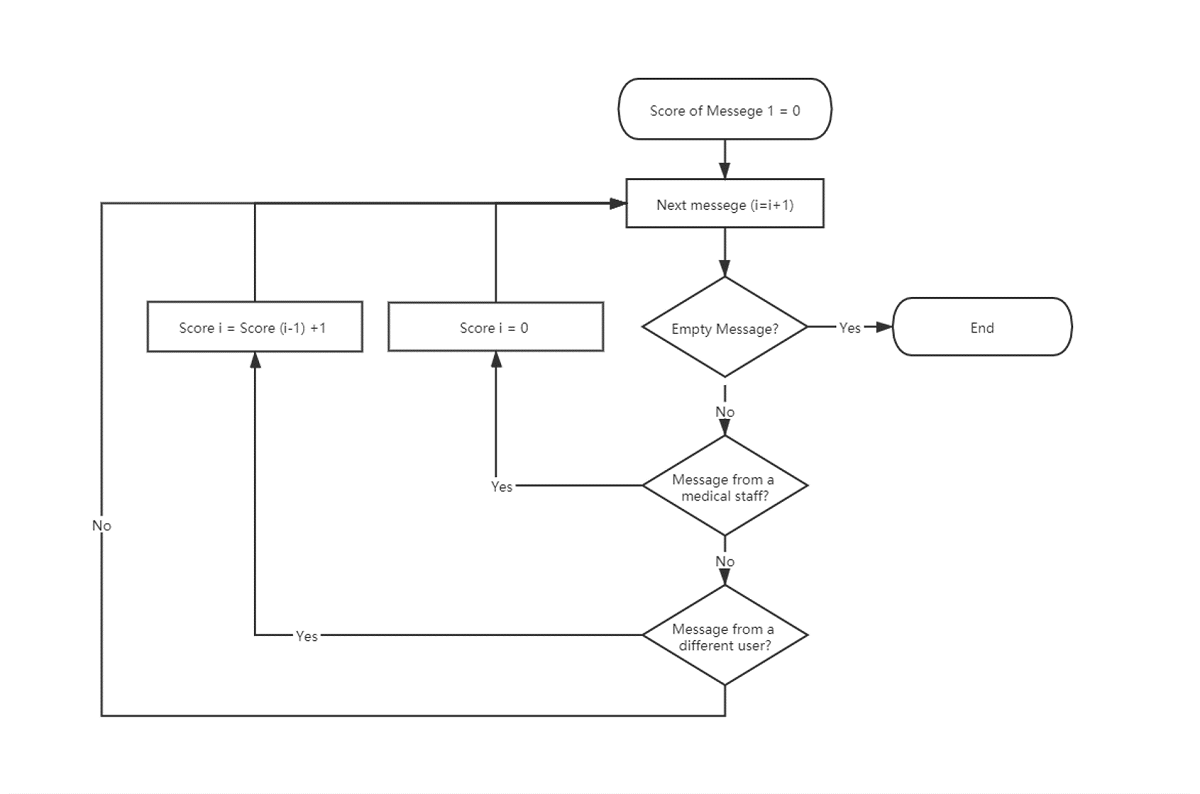

Supplement: Multimedia Appendix 3 [file mhealth_v9i3e18763_app3.png]
